# Female sex is associated with worse survival in laryngeal head and neck squamous cell carcinoma

**DOI:** 10.1093/oncolo/oyag167

**Published:** 2026-04-30

**Authors:** Alexandria G Yao, Amber Arif, Chloe A Paolucci, Sejal Jain, Joanna N Modi, Samyukta Mallick, Nadia Mezghani, Brian S Henick, Fatemeh Momen-Heravi, Alison M Taylor

**Affiliations:** Department of Pathology and Cell Biology, Columbia University Irving Medical Center, New York, NY, 10032, United States; Herbert Irving Comprehensive Cancer Center, Columbia University Irving Medical Center, New York, NY, 10032, United States; University of Kansas School of Medicine, Kansas City, KS, 66160, United States; Department of Pathology and Cell Biology, Columbia University Irving Medical Center, New York, NY, 10032, United States; Herbert Irving Comprehensive Cancer Center, Columbia University Irving Medical Center, New York, NY, 10032, United States; Department of Pathology and Cell Biology, Columbia University Irving Medical Center, New York, NY, 10032, United States; Herbert Irving Comprehensive Cancer Center, Columbia University Irving Medical Center, New York, NY, 10032, United States; Department of Pediatrics, University of Washington, Seattle Children’s Hospital, Seattle, WA, 98105, United States; Department of Pathology and Cell Biology, Columbia University Irving Medical Center, New York, NY, 10032, United States; Herbert Irving Comprehensive Cancer Center, Columbia University Irving Medical Center, New York, NY, 10032, United States; Department of Pathology and Cell Biology, Columbia University Irving Medical Center, New York, NY, 10032, United States; Herbert Irving Comprehensive Cancer Center, Columbia University Irving Medical Center, New York, NY, 10032, United States; Herbert Irving Comprehensive Cancer Center, Columbia University Irving Medical Center, New York, NY, 10032, United States; Division of Oral and Maxillofacial Surgery, Mount Sinai Health System, New York, NY, 10003, United States; Herbert Irving Comprehensive Cancer Center, Columbia University Irving Medical Center, New York, NY, 10032, United States; Herbert Irving Comprehensive Cancer Center, Columbia University Irving Medical Center, New York, NY, 10032, United States; Division of Periodontics, Department of Orofacial Sciences, University of California San Francisco, San Francisco, CA, 94143, United States; Department of Pathology and Cell Biology, Columbia University Irving Medical Center, New York, NY, 10032, United States; Herbert Irving Comprehensive Cancer Center, Columbia University Irving Medical Center, New York, NY, 10032, United States

**Keywords:** head and neck cancer, genomics, biological sex, laryngeal carcinoma

## Abstract

**Background:**

Despite treatment, approximately one-third of patients with head and neck squamous cell carcinoma (HNSCC) die within 5 years of diagnosis. Here, we assessed molecular and clinical features that correlate with biological sex and overall survival in HNSCC.

**Methods:**

We analyzed HNSCC cases in The Cancer Genome Atlas, which includes tumors from 382 males and 141 females. When possible, findings were validated with the National Cancer Institute’s Surveillance, Epidemiology, and End Results dataset (November 2023 Submission, 1975-2021).

**Results:**

Tumors from females had a significantly lower fraction of genome altered, along with fewer aneuploidy events. HNSCCs in females had increased expression of immune genes and higher overall immune infiltrate. Females showed worse overall survival in HNSCC compared to males (hazard ratio [HR]: 1.39). This disparity was statistically attributable to age at diagnosis as well as HPV status, as HNSCCs in females were less likely to be HPV-positive than in males. For laryngeal squamous cell carcinoma (L-HNSCC), females also had significantly worse outcomes (HR: 3.42), but here the disparity could not be attributed to available clinicogenomic features such as HPV or smoking status. The association of biological sex with outcome in L-HNSCC was also observed in the SEER database and was not present among patients diagnosed before the age of 50.

**Conclusion:**

Biological sex is associated with differences in survival and tumor genomic features in HNSCC. HPV infection status, diagnosis age, and tumor location contribute to a worse prognosis for females with HNSCC, particularly strong in L-HNSCC.

Implications for Practice Females with head and neck squamous cell carcinoma have poorer survival outcomes than males, in part due to lower rates of HPV. Using data from The Cancer Genome Atlas and the National Cancer Institute’s Surveillance, Epidemiology, and End Results database, this study demonstrated particularly strong sex differences between patients with laryngeal tumors, with females experiencing significantly worse survival outcomes. These findings suggest that biological sex may be an important consideration in risk stratification and clinical decision-making, especially for patients with laryngeal squamous cell carcinoma.

## Introduction

Head and neck squamous cell carcinoma (HNSCC) is a group of highly aggressive and biologically diverse tumors with an estimated 890,000 new cases and 450,000 deaths annually worldwide.^1^ HNSCCs can arise in multiple sites, including the lip, oral cavity, nasal cavity, paranasal sinuses, oropharynx, larynx, and nasopharynx.^2^ Primary tumor location has been shown to correlate with survival.^3^ Current treatments include surgery, radiation, and chemotherapy, with improved responses for HPV-positive tumors.^4^ However, overall 5-year survival remains around 68.5% in the United States.^5^

Biological sex is associated with clinical outcomes across many cancer types, with males often experiencing poorer survival.^6^ While HNSCC is more prevalent in males than females,^5^ some studies have reported lower overall survival among females with HNSCC.^7^ Genetic and hormone differences between the biological sexes can influence tumor progression, immunity, and therapeutic response,^8–11^ yet this has not been explored in HNSCC. Additionally, it is not known whether sex-associated survival differences vary by anatomic subsite within HNSCC.

To better define differences between male and female survival in HNSCC, we compared clinicogenomic features stratified by biological sex and evaluated their associations with survival. We found that biological sex correlates with overall survival, with the strongest disparity observed in larynx squamous cell carcinomas (L-HNSCCs).

## Methods

### Datasets

This study utilizes the HNSCC cohort from The Cancer Genome Atlas (TCGA),^12^ which includes the following data: biological sex (total *n* = 521), stage (521), HPV status (469), tumor location (521), mutation count (521), copy number alterations (512), aneuploidy (509), immune gene set expression (510), and survival (521). For L-HNSCC, additional data was analyzed from National Cancer Institute’s Surveillance, Epidemiology, and End Results (SEER) dataset (November 2023 Submission, 1975-2021).^3^ Survival data were specifically pulled for patients diagnosed from 2000 to 2020, with a total of 13,793 females and 57,595 males.

### Statistical methods

Univariable and multivariable Cox proportional hazards regression with different covariates was performed using the Survival package and coxph() function in R. Survival curves were statistically compared via a log-rank test. Differential gene expression analysis between biological sexes was performed using DESeq2.^13^ Significantly enriched pathways and genes were identified using the gene set enrichment analysis (GSEA) algorithm.^14^ For genome- or transcriptome-wide analyses, *P*-values were adjusted using the Benjamini–Hochberg (BH) correction, with corrected *P*-value less than .05 considered significant. Student’s *t*-test was utilized for comparing 2 groups, with *P*-value less than .05 considered significant.

## Results

### Genomic features of HNSCC by biological sex

**Figure 1.**
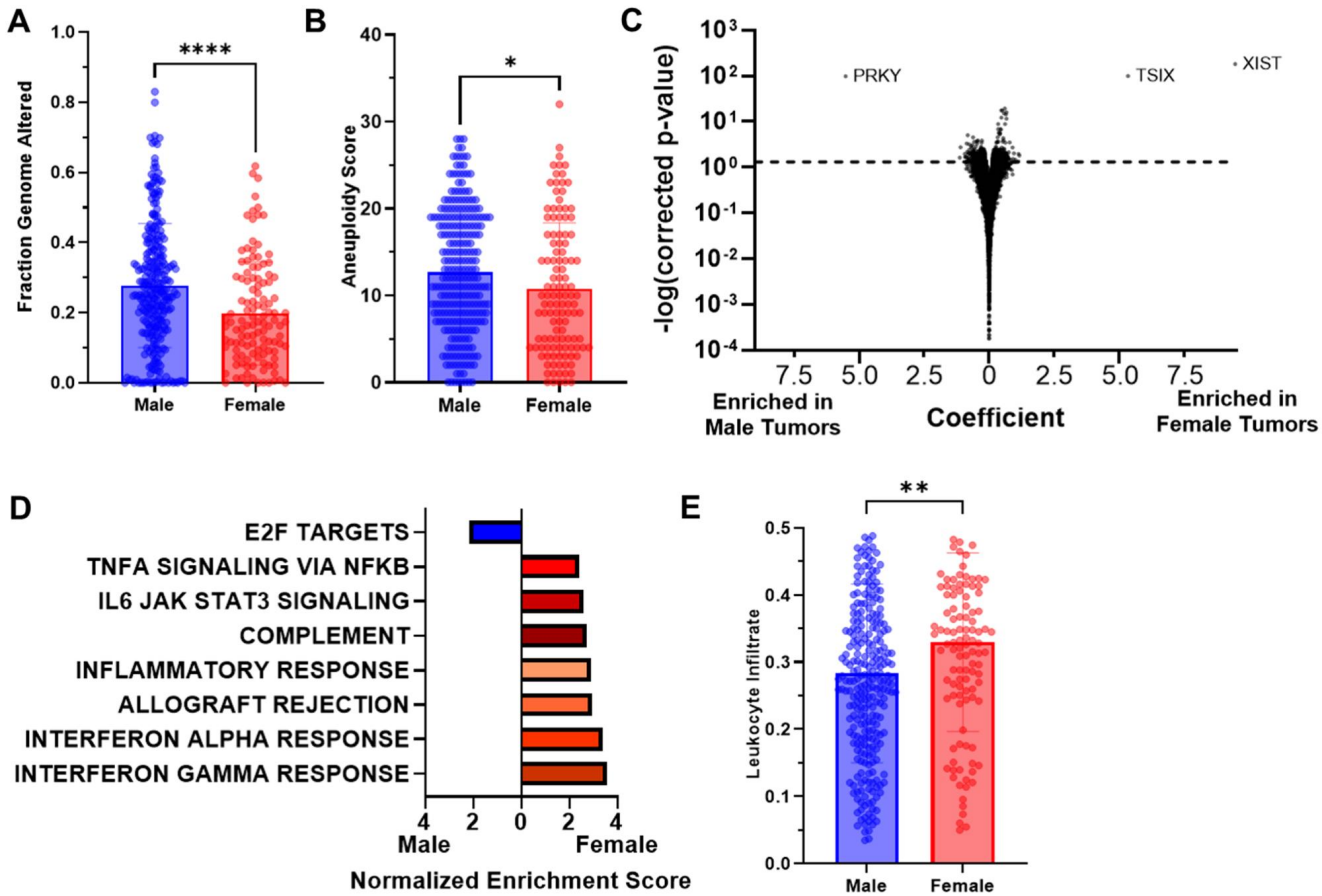
**Molecular correlates with biological sex**. HPV-negative tumors in the TCGA HNSCC cohort (*n* = 393-396). Stratified by biological sex: (A) fraction of genome altered (Student’s *t*-test *P* < .0001) and (B) aneuploidy score (Student’s *t*-test *P*-value = .0159). (C) Volcano plot of statistical significance (y-axis = -log of the false-discovery-rate-corrected *P*-value) by biological sex enrichment for gene expression. (D) Normalized enrichment score for differential pathways identified by gene set enrichment analysis between male and female, FWER *P*-values * <.05; ** < .01; *** < .001. Stratified by biological sex (*n* = 394): (E) leukocyte infiltrate (Student’s *t*-test *P*-value = .0016). *P < .05, **P < .01, ****P < .0001.

We analyzed 521 HNSCC samples from the TCGA, 381 from males and 140 from females (Table 1). To eliminate HPV as a confounding factor, genomic and transcriptomic analyses focused on the HPV negative subset (*n* = 415, 292 males and 123 females). We found no significant difference in mutation count between tumors from males or females. Rather, tumors in males had a higher fraction of genome altered (*P*-value < .0001; Figure 1A) and a higher aneuploidy score (*P*-value = .0159; Figure 1B), consistent with other analyses of this data.^15^

**Table 1.** **Clinical characteristics of HNSCCs in the Cancer Genome Atlas**.

Variable	Male (*n* = 381)	Female (*n* = 140)
**Age at diagnosis mean (SD)**	59.5 (10.8%)	64.6 (13.4%)
**Age at diagnosis range**	19-87	24-90
**Smoking status**		
** Never**	68 (17.8%)	54 (38.6%)
** Former**	193 (50.7%)	53 (37.9%)
**Current**	111 (29.1%)	27 (19.3%)
** Unavailable**	9 (2.4%)	6 (4.2%)
**Tumor location**		
** Oral cavity (including tongue, alveolar ridge, floor of mouth, lip, and buccal mucosa)**	232 (60.9%)	108 (77.1%)
** Oropharynx/tonsil**	45 (11.8%)	9 (6.4%)
** Larynx/hypopharynx**	104 (27.3%)	23 (16.4%)
**Overall survival (months)**	30.3	29.4
**Progression-free survival (months)**	27.0	27.0
**HPV status**		
** Negative**	277 (72.7%)	119 (85%)
** Positive**	68 (17.8%)	5 (3.6%)
** Not called/unavailable**	23 (6.0%)	13 (9.2%)
**Tumor grade**		
** T0**	1 (0.3%)	0 (0%)
** T1**	33 (8.7%)	20 (14.3%)
** T2**	103 (27.0%)	41 (29.3%)
** T3**	73 (19.2%)	25 (17.9%)
** T4 (a, b)**	137 (36.0%)	47 (33.6%)
**Unavailable**	34 (8.9%)	7 (5%)
**Histological grade**		
** G1**	41 (10.8%)	21 (15%)
** G2**	219 (57.5%)	91 (65%)
** G3**	99 (26.0%)	25 (17.9%)
** G4**	7 (1.8%)	0 (0%)
**Unavailable**	15 (3.9%)	3 (2.1%)
**Lymph node**		
** N0**	121 (31.8%)	56 (40%)
** N1**	54 (14.2%)	17 (12.1%)
** N2 (A, B, C)**	145 (38.1%)	43 (30.7%)
** N3**	6 (1.6%)	2 (1.4%)
**Unavailable**	55 (14.4%)	22 (15.7%)

Next, we looked at specific mutation and copy number patterns. For mutations, no gene passed multiple-hypothesis correction to be significantly correlated with biological sex. Tumors from females showed higher copy number for several 3p loci (Figure S1A) or the entire chromosome arm (Figure S1B), representing less frequent deletion in these individuals. 3q11.1 gain, 9p deletion, and 11q deletion were more frequent in tumors from males, both consistent with the overall higher levels of copy number alterations (Figure S1A,B). In contrast, tumors from females showed more frequent 9q24.1 amplification and 2q deletion (Figure S1A,B).

### Transcriptomic HNSCC features by biological sex

Gene expression analysis of HPV-negative tumors identified top differentially expressed genes including *XIST* and *TISX* (located on the X chromosome, higher in females) and *PRKY* (located on the Y chromosome, higher in males) (Figure 1C). GSEA^14^ found HPV-negative tumors in males were significantly enriched for expression of E2F targets (normalized enrichment score [NES] = 2.2, FWER *P*-value = .014). HPV-negative tumors in females showed enrichment for pathways involved in interferon gamma response (NES = 3.6), interferon alpha response (NES = 3.4), allograft rejection (NES = 2.9), inflammatory response (NES = 2.9), and hallmark complement (NES = 2.7) (all FWER *P*-value < .001) (Figure 1D).

We compared immune cell type fractions estimated from gene expression data using xCell.^16^ Many immune cell types were significantly higher in the tumors of females, including dendritic cells (*P*-value = .0028; Figure S1C), monocytes (*P*-value = .0003; Figure S1D), and regulatory T cells (*P*-value = .0002; Figure S1E). Using immune infiltrate estimates measured by methylation patterns, we found higher overall leukocyte fraction in tumors from females (*P*-value = .0016, Figure 1E). These findings align with the extensive literature that females have higher innate immune signaling, including in tumors.^17^

We hypothesized that the increased immune infiltrate contributed to the increased expression of immune genes in the tumor sample, as the tumor sample is not pure and contains stromal populations and immune cells in addition to tumor cells. To test this, we created a linear model to assess gene expression correlation. In the univariate model, just looking at biological sex, we found several immune and keratinization gene ontology groups enriched in the tumors of females (Figure S1F). When leukocyte fraction and age were added to the linear regression, sex no longer significantly correlated with most immune pathways (Figure S1F). If we instead added a variable for tumor location (grouped into oral cavity/tongue, tonsil/oropharynx, and larynx/hypopharynx), the keratinization pathway was no longer significant (Figure S1F). These data indicate that the differential gene expression pathways between males and females may be related to the tumor location and general immune infiltrate.

### Clinical features of HNSCC by biological sex

**Figure 2.**
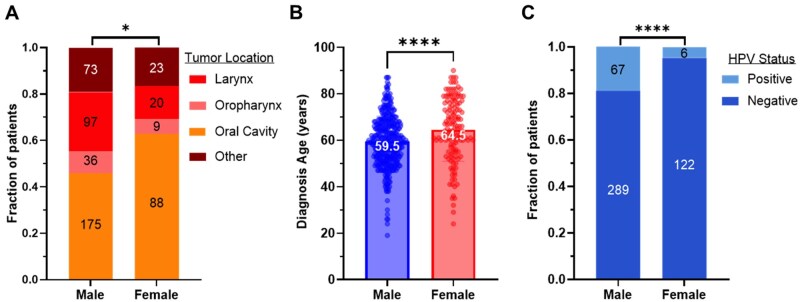
**Clinical correlates with biological sex**. TCGA HNSCC cohort of 521 samples. The following variables are stratified by biological sex: (A) Tumor location subtype distribution (chi-square *P*-value = .046), (B) diagnostic age (Student’s 2-tailed *t*-test, *P*-value <.0001), and (C) HPV distribution among males and females (*n* = 484, Fisher’s exact *P*-value <.0001). *P < .05, ****P < .0001.

We next wanted to assess clinical features that correlate with biological sex in the TCGA dataset. Distribution of tumor location varied between biological sexes (*P*-value = .03), with females having a greater proportion of oral cavity tumors (*P*-value = .0373) (Figure 2A). Tumor and lymph grade did not differ significantly between populations (Figure S2A and B). However, diagnosis age was significantly different, with mean at 59.5 and 64.5 years for males and females, respectively (Figure 2B, *P*-value < .0001). Females had a lower frequency of HPV-positive tumors than males (67/356 for male, 6/128 for female, *P*-value < .0001; Figure 2C), consistent with other cohorts.^2^ Though not statistically significant, a higher proportion of males in this cohort were current or former smokers, while a higher proportion of females had never smoked (Figure S2C).

**Figure 3.**
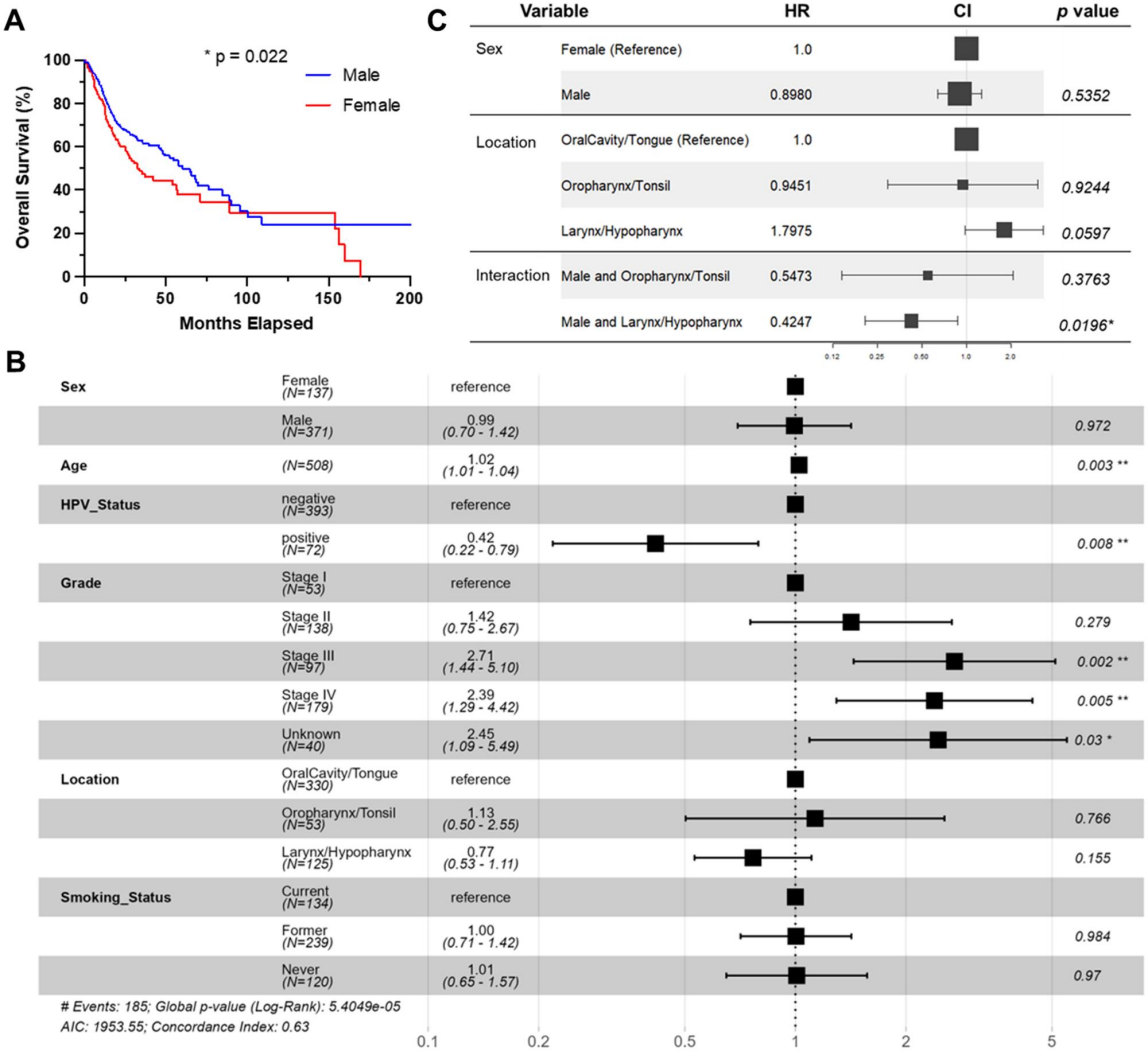
**Survival correlations with biological sex**. (A) Overall survival stratified by biological sex, HR: 1.39, 95% CI: 1.02-1.88, *P*-value = .022. (B, C) Forest plots displaying hazard ratios and confidence intervals from multivariable regression model for HNSCC survival; (B) model with sex, age, HPV status, grade, location, and smoking status; (C) model with sex, tumor location, and interaction of these 2 variables. *P < .05, **P < .01.

Consistent with previous studies,^7^ females with HNSCC had worse overall survival outcomes than males in this cohort, with a median survival of 32.8 months for females and 60.4 months for males (Figure 3A, hazard ratio [HR]: 1.39, 95% CI: 1.02-1.88, *P*-value = .022). We performed survival analysis for 5 additional variables (Figure S3A). Increased age at diagnosis significantly correlated with worse overall survival (HR: 1.023, 95% CI: 1.01-1.036, *P*-value = .000495). HPV-positivity correlates with better overall survival within HNSCC,^18^ and we observed the same in this dataset (HR: 0.44, 95% CI: 0.26-0.74, *P*-value = .002). More advanced stage at diagnosis (“Grade”) had worse survival (Stage II HR: 1.2, Stage III HR: 2.2, Stage IV HR: 2.0). Smoking history, grouped into former, current, and never smoker, did not show significance in the model. For tumor location, samples were split into 3 tumor location groups: oral cavity/tongue (oral cavity, tongue, alveolar ridge, buccal mucosa, floor of mouth, hard palate, and lip, *n* = 185); tonsil/oropharynx (*n* = 54); and larynx/hypopharynx (*n* = 127). Using oral cavity/tongue as reference, oropharynx/tonsil showed significantly improved survival (HR: 0.56, 95% CI: 0.32-0.99, *P*-value = .048). Larynx/hypopharynx was not significantly different from oral cavity/tongue.

We next performed multivariable analysis for all 6 variables combined (Figure 3B). Sex and tumor location were no longer significant. Age, HPV status, and tumor grade remained significant. We next paired each variable with biological sex for the model (Figure S3B). Sex remained significant in a model with tumor grade but not HPV and age, suggesting that the sex survival disparity is in part driven by differences in HPV status and age at diagnosis. Interestingly, in a model with tumor location, sex, and an interaction variable, we observed a statistically significant interaction in larynx/hypopharynx tumors, with these tumors showing improved survival in males (Figure 3C).

### Laryngeal cancer and outcomes by biological sex

**Figure 4.**
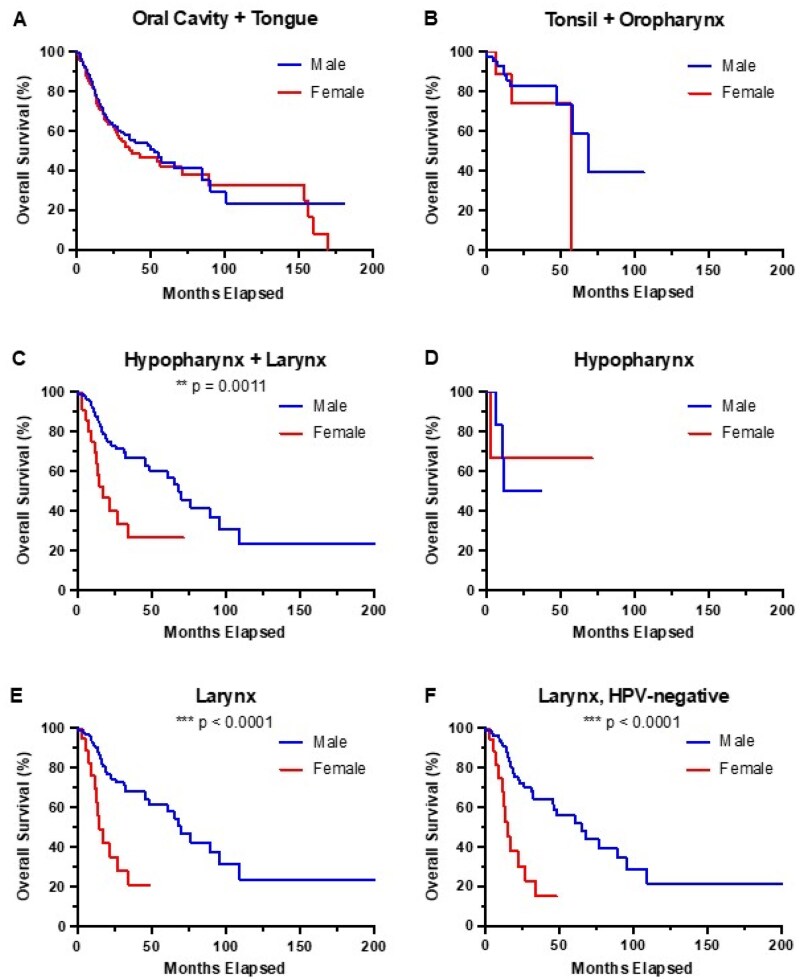
**HNSCC of the larynx shows significantly worse survival outcomes in female patients**. Overall survival stratified by biological sex for the following tumor locations, with *P*-values calculated from the log-rank Mantel-Cox test: (A) oral cavity and tongue tumors, *P*-value = .4442 (*n* = 340), (B) tonsil and oropharynx, *P*-value = .3050 (*n* = 54), (C) hypopharynx and larynx, *P*-value = .0011 (*n* = 127), (D) hypopharynx, *P*-value = .8522 (*n* = 10), (E) larynx, *P*-value < .0001 (*n* = 117), (F) larynx, HPV-negative, *P*-value <.0001 (*n* = 99). **P < .01, ***P < .001.

We next wanted to assess whether tumor location impact the sex disparity in outcomes. Samples were split into the same 3 tumor location groups. Patients with tumors in the oral cavity/tongue (Figure 4A) and tonsil/oropharynx (Figure 4B) did not show a significant difference between male and female survival (oral cavity/tongue HR: 1.14, 95% CI: 0.81-1.59, *P*-value = .44; tonsil/oropharynx HR: 1.92, 95% CI: 0.39-9.42, *P*-value = .31). In contrast, tumors in the hypopharynx and larynx significantly differ in survival outcomes between males and females (Figure 4C, HR: 2.69, 95% CI: 1.13-6.42, *P*-value = .0011). Within this group, patients with hypopharynx tumors did not show a significant survival outcome difference when comparing between biological sex (Figure 4D, HR: 0.81, 95% CI: 0.09-6.96, *P*-value = .852), while larynx tumor patient survival shows a significant difference with worse survival in females (Figure 4E, HR: 3.42, 95% CI: 1.26-9.26, *P*-value < .0001). HPV-associated L-HNSCC is thought to be very low^19^ and not a driver in this anatomical subsite.^20^^,^^21^ However, in our dataset, 5/110 laryngeal tumors were HPV-positive. This does not affect the survival disparity; patients with HPV-negative L-HNSCC showed a similar significant difference when comparing biological sex survival outcomes (Figure 4F, HR: 3.60, 95% CI: 1.31-9.93, *P*-value < .0001). In L-HNSCC, females had significantly worse survival regardless of HPV status.

We wanted to validate this finding with alternative data set and turned to the National Cancer Institute’s SEER*Explorer.^3^ Here, we looked at the relative survival rates of individuals with larynx cancer of all diagnosis ages from years 2000-2020, with a total of 13,793 females and 57,595 males. Between males and females of all ages, males again had better survival rates compared to females (Figure S4A, *P*-value < .00001). Due to the large size of this cohort, we were able to stratify survival by age at diagnosis. When diagnosed before 50 years of age, females with L-HNSCC exhibited better survival outcomes, although not statistically significant (Figure S4B, *P*-value = .2593). However, for males and females between the ages of 50-64 and 65+, males had better survival (Figure 4C and D, *P*-value = .0002 and *P*-value < .00001, respectively). For both females and males, diagnosis before 65 showed better overall survival than diagnosis 65+ (Figure S4E, *P*-value = .00001; Figure S4F, *P*-value <.00001). Overall, these findings are consistent with our findings in the smaller TCGA cohort and consistent with other pan-cancer and larynx/oropharynx analyses of SEER data^6^ and mortality analyses.^22^

### Clinicogenomic characteristics by biological sex in L-HNSCC

We next investigated whether any clinicogenomic features correlated with biological sex in L-HNSCC. Overall alteration frequency (mutations, copy number, and aneuploidy) was not significantly different between males and females. No individual gene mutations were significantly different after correction for multiple hypotheses and overall mutation rate. Controlling for fraction genome altered, aneuploidy trends were similar to pan-HNSCC analyses. Uniquely in L-HNSCC, 11q is significantly more frequently deleted in males (Figure S5A). When analyzing gene expression correlations within L-HNSCC, we again observed that *PRKY*, *TSIX*, and *XIST* were the most significantly differential genes in males vs females (Figure S5B). In gene ontology (GO) pathway correlations, tumors from males showed significant enrichment of oxidative phosphorylation, myogenesis, and hypoxia pathways (Figure S5C, FWER *P*-values for each pathway: <.0001, .002, and .093, respectively). The minimal differences observed here suggest that non-genomic factors may play a larger role in the L-HNSCC biological sex disparity.

**Figure 5.**
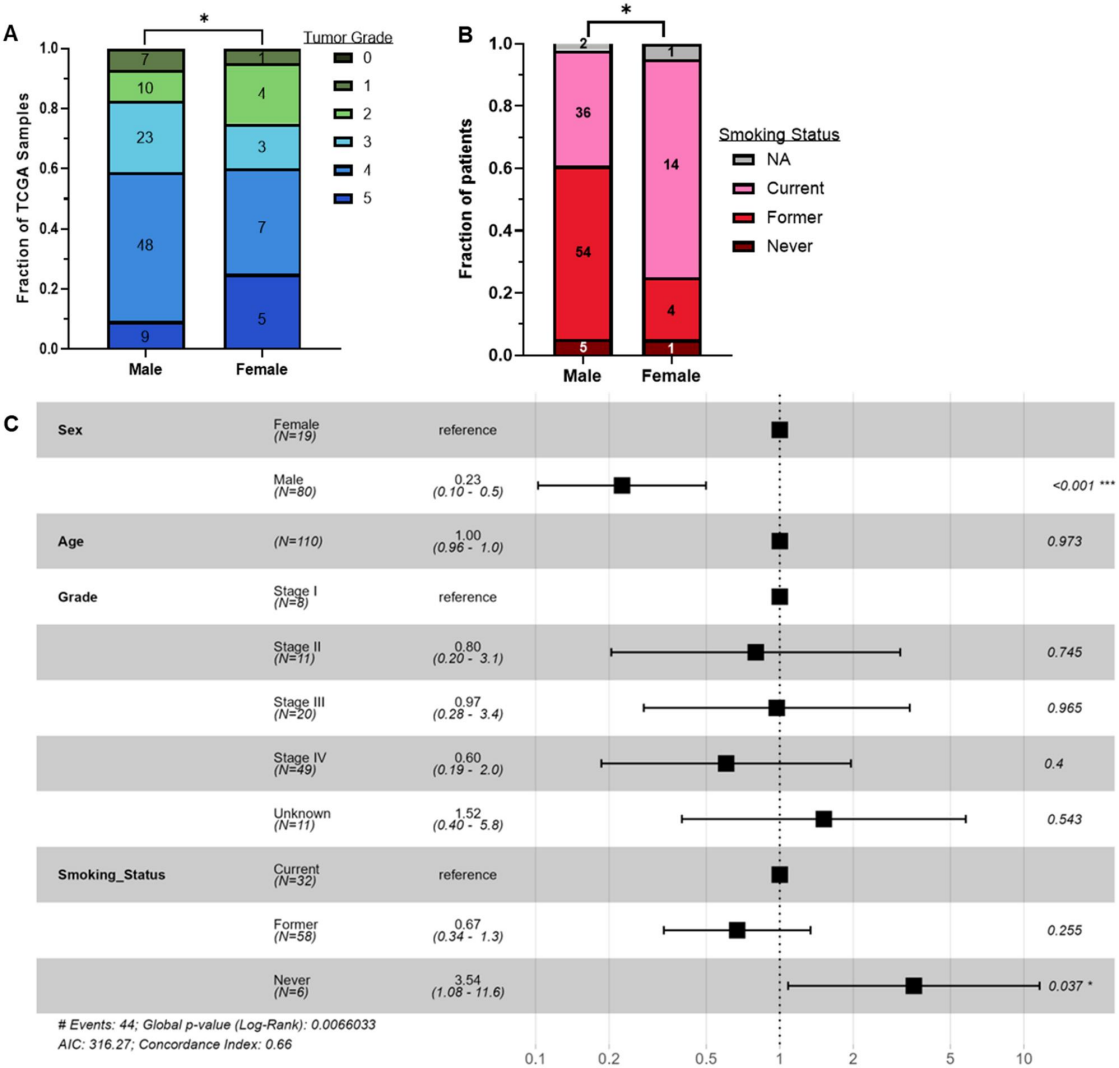
**Clinicogenomic data stratified by biological sex in L-HNSCC**. The following variables are stratified by biological sex (*n* = 117, males = 97 and females = 20): (A) tumor grade (chi-square *P*-value = .03) and (B) smoking status (chi-square, *P*-value = .0135). (C) Forest plot displaying hazard ratios and confidence intervals from multivariable regression model for L-HNSCC. *P < .05, ***P < .001.

Clinically, we found that L-HNSCC patients showed a significant difference in tumor grade distribution by sex, with higher tumor grade in males (*P*-value = .03, Figure 5A). Lymph grade stage 1 or higher is more frequent in females, though not significant (*P*-value = .068). Similarly, diagnostic age did not differ significantly by sex within L-HNSCC (*P*-value = .4149). In contrast to analyses across all HNSCC tumor locations (Figure S2C), a higher proportion of females with L-HNSCC were current smokers (*P*-value = .0135, Figure 5B).

Lastly, within the 110 L-HNSCC samples in TCGA, we generated a multivariable survival model with 4 variables—sex, age, tumor grade, and smoking status (Figure 5C). (Only 5 L-HNSCC samples were HPV-positive, so we did not include this as a variable.) In the multivariable model, males still had significantly better survival (HR: 0.23, 95% CI: 0.1-0.5, *P*-value < .001). Neither age nor grade was significant, but never smokers (*n* = 6) had a significantly worse outcome. Overall, these analyses again suggest that non-genomic factors contribute to the biological sex disparity in L-HNSCC.

## Discussion

In HNSCC, females have worse survival outcomes than males. Our results highlight the differences between male and female clinical, transcriptomic, and genomic characteristics. We found that in addition to age and HPV status, tumor location (particularly larynx) was a driving factor for survival differences between biological sexes with HNSCC.

In our genomic analyses of the tumors, we observed significant differences between males and females for copy number alterations but not mutations. HPV-negative HNSCCs in males had higher aneuploidy and fraction genome altered, and most individual copy number alterations occurred more frequently in tumors from males. Interestingly, there were some alterations that occurred in more frequently in tumors from females, including gain of 9q24.1 and deletion of several loci on 2q. These 2q loci include COSMIC Tier 1 cancer genes such as *ACKR3* and *CXCR4*, which both encode chemokine receptors. It would be interesting to follow up on the role of these genes in HNSCC. Overall, our data suggest that there are only a few somatic differences in the genomics of HPV-negative HNSCC patients of different biological sex.

Consistent with work in healthy tissues,^17^ females have higher expression of genes in immune signaling pathways and higher immune infiltrate in HNSCC, independent of HPV status. These data would suggest that biological sex might affect response to immunotherapy. Interestingly, we found enrichment of immune-related pathways in HNSCCs from females, including interferon signaling. Changes in interferon expression have been reported to influence immunotherapy response.^23^ Future studies should assess how biological sex predicts response to immunotherapy in HNSCC.

Our transcriptomic analysis also identified pathways up-regulated in tumors from males, most significantly E2F target genes. Genes in this pathway are involved in cell cycle regulation, and their up-regulation is consistent with increased proliferation. This, as well as the higher load of genomic alterations, are both generally expected to correlate with more aggressive tumors and worse outcomes. Our findings show that instead females have worse outcomes, again suggesting non-molecular factors at play.

Beyond HPV and diagnosis age, tumor location is a significant driver of sex disparity. In the larynx, SCC is associated with smoking and alcohol use but less frequently with HPV infection.^19^ Incidence is much higher in males, and overall mortality is high, particularly for late-stage disease.^22^ Although we describe a survival sex disparity for L-HNSCC, the cause of this disparity remains unknown. For L-HNSCC, analyses of the large SEER database suggest that females under 50 may actually have improved survival; this is consistent with studies of nasopharyngeal carcinoma showing pre-menopausal benefits in survival,^24^ which is postulated to be due to hormonal differences. Loss of estrogen receptor is associated with more aggressive tumors in xenografts of laryngeal cancer cell lines.^25^ A larger dataset with information about the estrogen receptor protein and pathway, as well as menopausal state, would be needed to test this hypothesis.

Although there is some clinical information in the TCGA dataset, it is not universal. For example, we observed a higher frequency of current smokers among females with L-HNSCC; although this increased frequency did not seem to be driving our observed survival differences, 2.5% of L-HNSCCs did not have smoking data. Tumor location within the larynx (glottal, supraglottal, subglottal) has also been shown to affect patient outcomes.^26^ One study suggested that this may be driving the observed disparity in L-HNSCC.^27^ Others have tried stratifying TCGA data by these locations^28^; this did not seem to explain our survival differences but still remains a possibility. We also do not know if our findings are specific to US populations. Our analyses of SEER data are consistent with some studies,^6^ and a study in England and Wales has similar findings as well.^29^ In contrast, studies in Israel and China show different correlations.^30^^,^^31^ These geographic differences could be due to environmental differences or race/ancestry differences. Future studies looking at the interaction between biological sex and ancestry in outcomes will be critical. Overall, more patients with clinical and genomic data points for each tumor location of the HNSCC patient population would greatly strengthen these analyses.

## Conclusion

There are differences in overall survival between males and females with HNSCC, impacted by diagnosis age, tumor site, and HPV infection status. Disparities in L-HNSCC prompt inquiry around clinical interventions, such as earlier screening for females with certain lifestyle indicators or adjusting treatment approaches for this patient population.

## Supplementary Material

oyag167_Supplementary_Data

## Data Availability

The raw TCGA data, processed data, and clinical data can be found at the legacy archive of the GDC (https://portal.gdc.cancer.gov/legacy-archive/search/f) and the PanCanAtlas publication page (https://gdc.cancer.gov/about-data/publications/pancanatlas). The copy number data can be found here: (https://gdc.cancer.gov/about-data/publications/pancan-aneuploidy). The mutation data can be found here (https://gdc.cancer.gov/about-data/publications/mc3-2017). The gene expression data can be found here (https://gdc.cancer.gov/about-data/publications/pancanatlas). TCGA data can also be explored through the Broad Institute FireBrowse portal (http://gdac.broadinstitute.org) and the Memorial Sloan Kettering Cancer Center cBioPortal (http://www.cbioportal.org).
