# Supplementary material for: Female sex is associated with worse survival in laryngeal head and neck squamous cell carcinoma
Source: Oncologist. 2026 Apr 30;31(6):oyag167. doi: 10.1093/oncolo/oyag167 (PMC13192479; doi:10.1093/oncolo/oyag167)
Supplement: oyag167_Supplementary_Data [file oyag167_supplementary_data.pdf]

Supplemental Figures

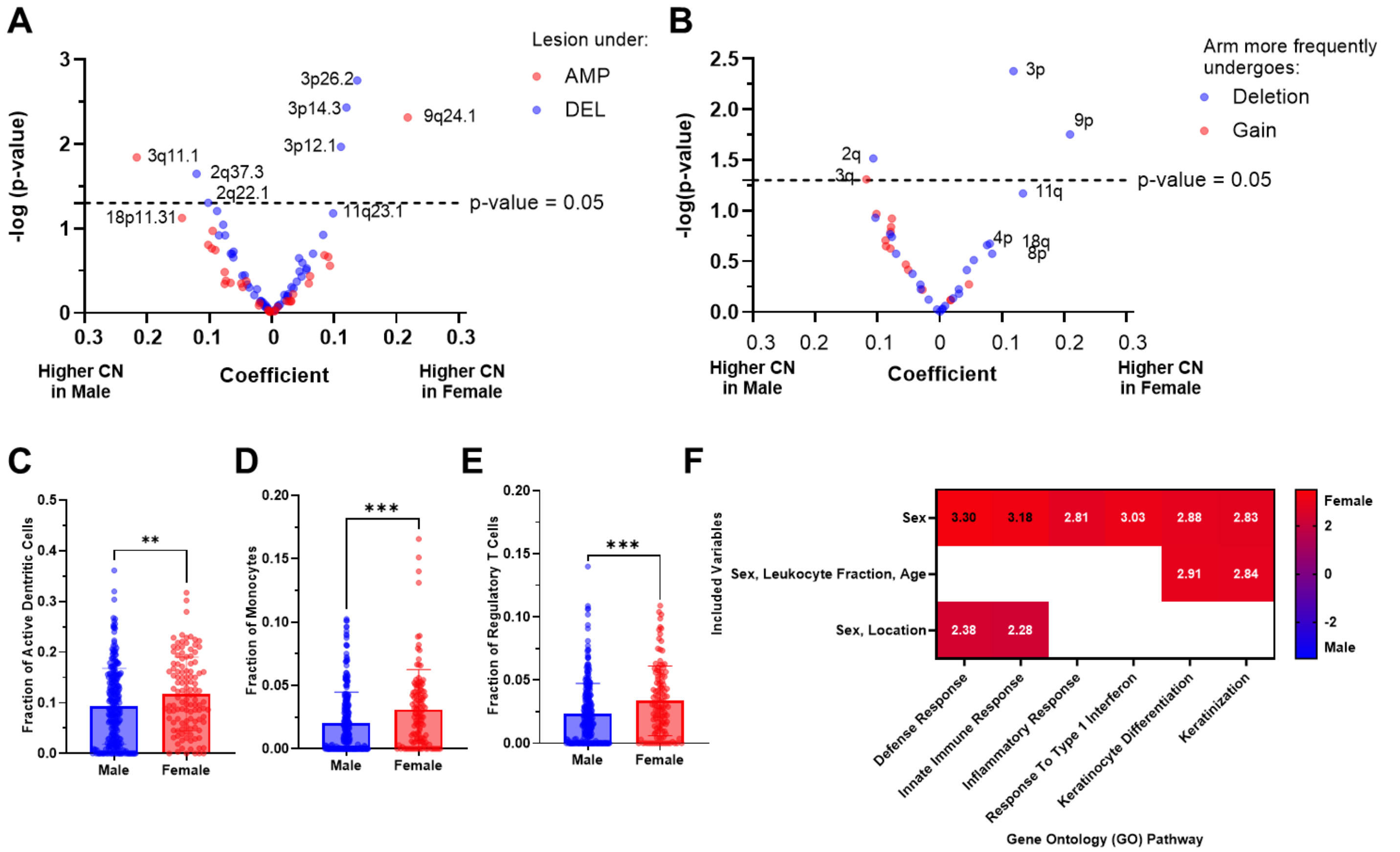

Supplemental Figure 1. Genomic and transcriptomic correlates with biological sex.
(a-b) Volcano plot of statistical significance (y-axis) by biological sex enrichment for (a) copy number lesions, and (b) aneuploidy events.
(c-e) Stratified by biological sex (n= 394): (d) fraction of active dendritic cells, (e) fraction of monocytes, and (f) fraction of regulatory t-cells.
(f) Heat map represents normalized enrichment scores based on linear modeling of gene expression correlations with biological sex. Additional variables include leukocyte fraction and age (middle row) or tumor location (bottom row).

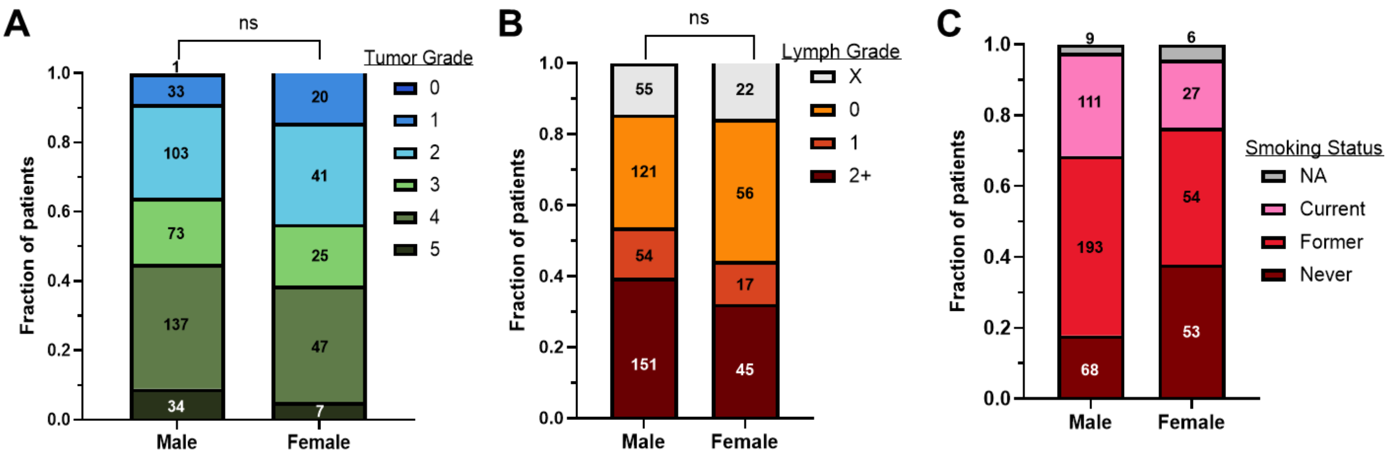

Supplemental Figure 2. HNSCC clinical features by biological sex.
The following variables are stratified by biological sex: (a) tumor grade (Chi-square p-value = 0.3063), (b) lymph grade (Chi-square p-value = 0.143), (c) smoking history (Chi-square p-value = 0.1012)

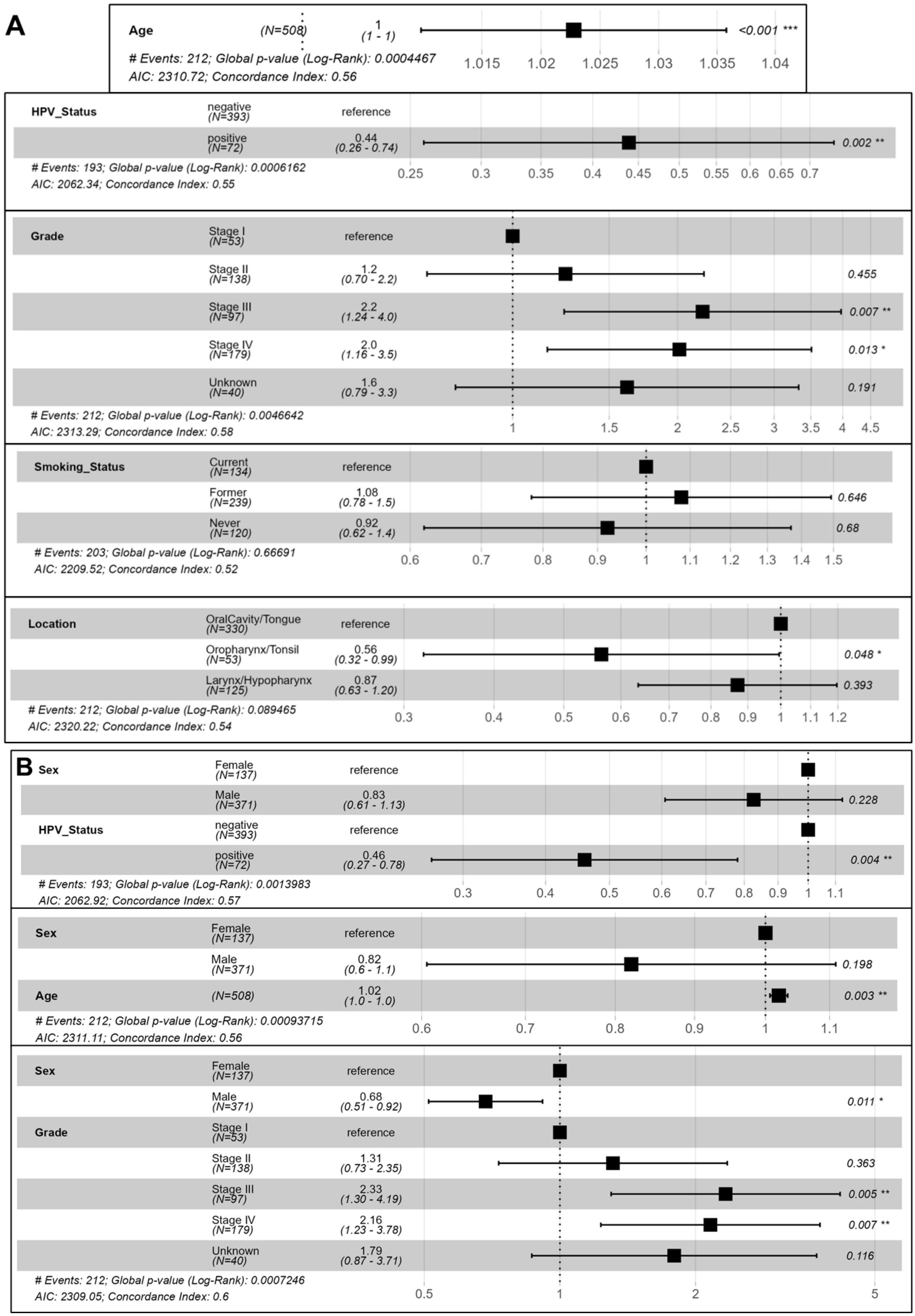

Supplemental Figure 3. Survival regression models for HNSCC. Forest plots displaying hazard ratios and confidence intervals from (a) univariable regression model and (b) sex with one additional variable.

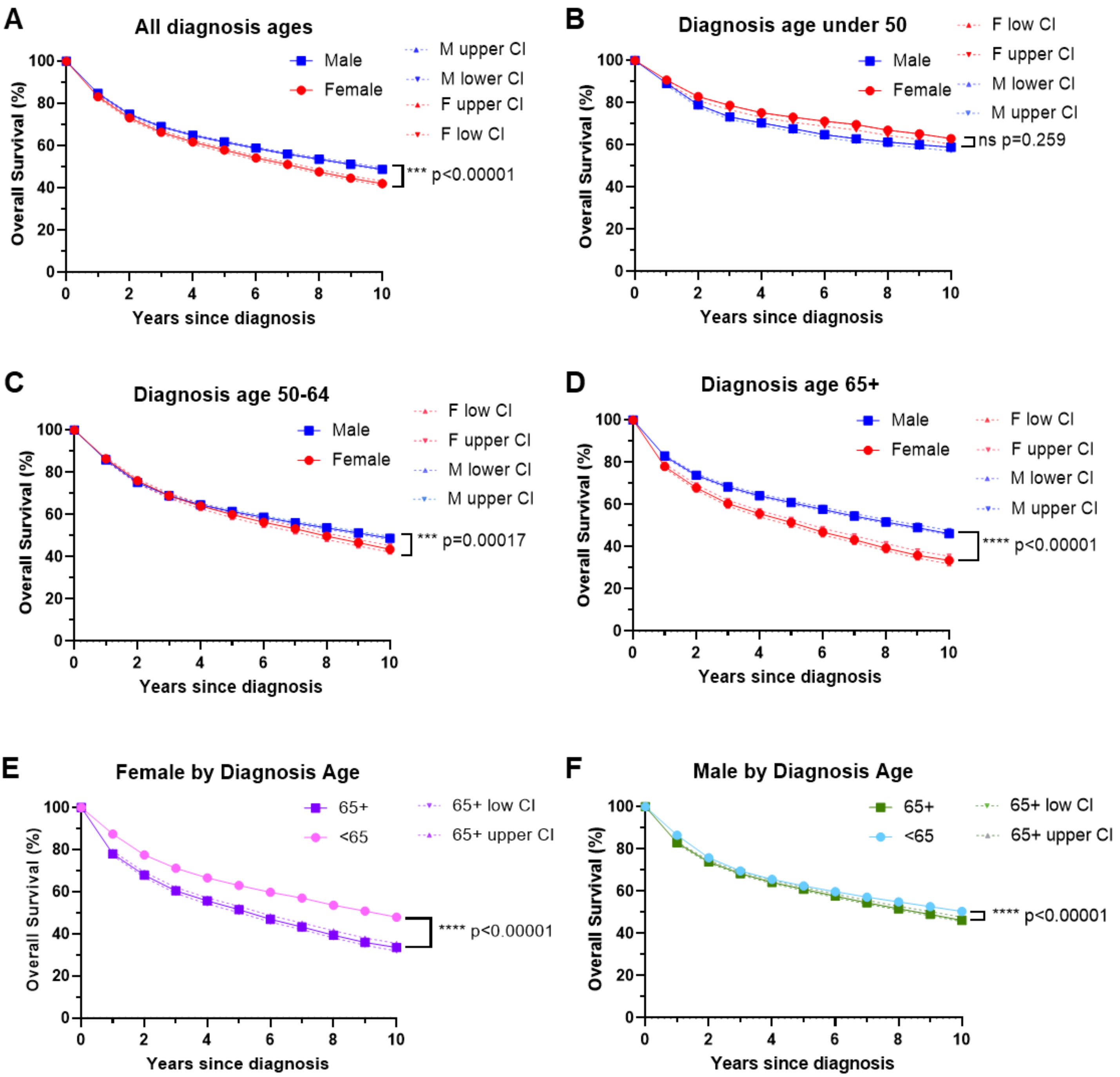

Supplemental Figure 4. Relative survival by biological sex in a SEER cohort of L-HNSCCs.
Overall survival stratified by biological sex and age groups, with p-values calculated from the log-rank Mantel-Cox test:
a, All ages, two-tailed paired p-value = 0.0002 (***).
b, Under 50 years old, p-value = 0.259 (ns).
c, 50-64 years old, p-value = 0.00017 (***).
d, 65+ years old, p-value = 0.00001 (****).
e, Female: under 64 vs 65+ years old, p-value < 0.00001 (****).
f, Male: under 64 vs 65+ years old, p-value < 0.00001 (****).

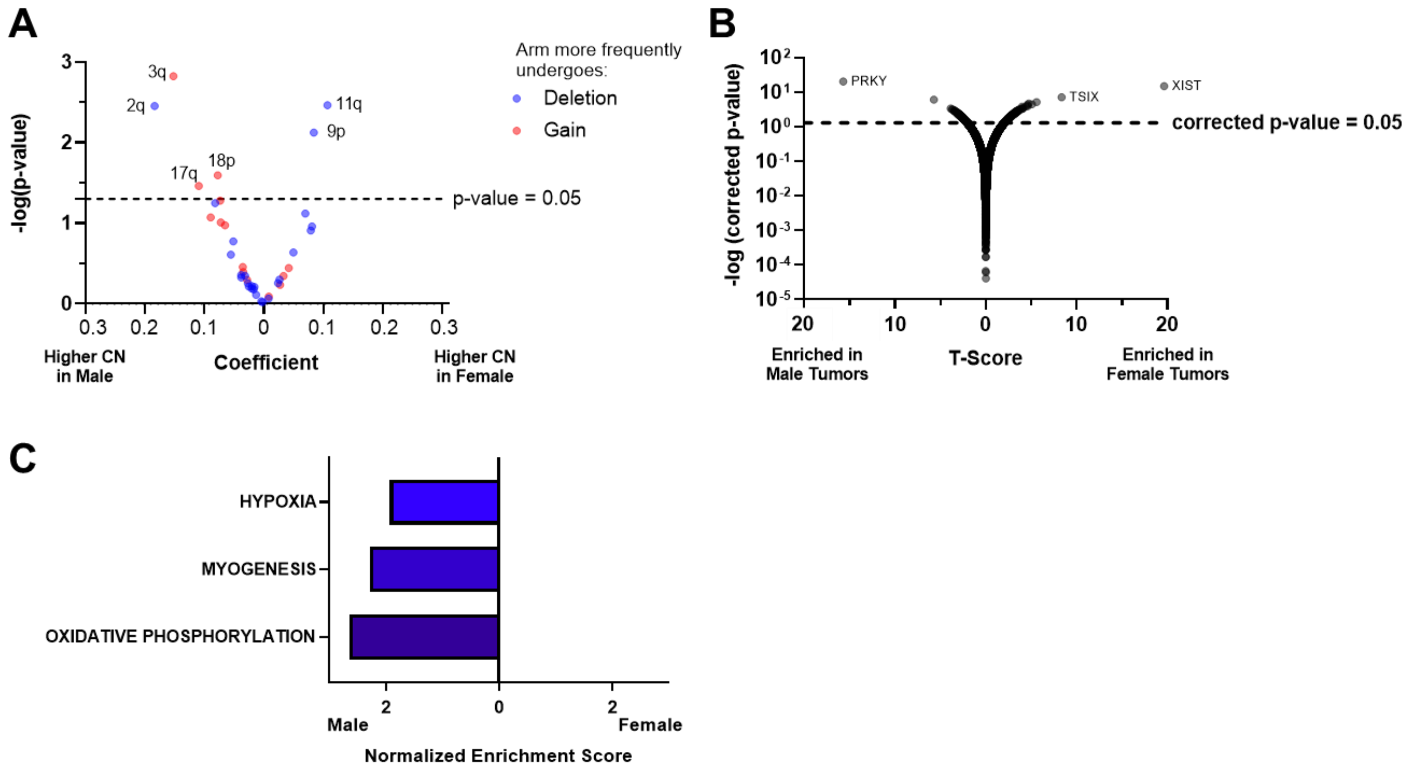

Supplemental Figure 5. Genomic and transcriptomic correlates with biological sex in L-HNSCC. TCGA L-HNSCC within the HNSCC cohort. Volcano plot of statistical significance (y-axis) by biological sex enrichment for (a) aneuploidy events and (b) gene expression. (c) Pathway analysis of gene expression correlates with biological sex. FWER values from bottom to top: <0.0001, 0.002, and 0.093.
